# Long-term follow-up of protective effects on salivary and swallowing structures and improvement of late xerostomia and dysphagia by level IIb optimisation in clinical target volume of nasopharyngeal carcinoma

**DOI:** 10.1186/s12885-024-12391-7

**Published:** 2024-05-27

**Authors:** Jiawei Zhou, Li Wang, Ting Qiu, Han Gao, Lijun Wang, Shengfu Huang, Xia He, Lirong Wu

**Affiliations:** Department of Radiation Oncology, Jiangsu Cancer Hospital & Jiangsu Institute of Cancer Research & The Affiliated Cancer Hospital of Nanjing Medical University, Nanjing, 210009 China

**Keywords:** Nasopharyngeal carcinoma (NPC), Xerostomia, Dysphagia, Clinical target volume (CTV), Long-time survival

## Abstract

**Background:**

This study aimed to assess the long-term effect of level IIb clinical target volume (CTV) optimisation on survival, xerostomia, and dysphagia in patients with nasopharyngeal carcinoma (NPC).

**Methods:**

Clinical data of 415 patients with NPC treated with intensity-modulated radiotherapy between December 2014 and October 2018 were retrospectively analysed. The patients were categorised into modified and comparison groups. Late xerostomia and dysphagia were evaluated using Radiation Therapy Oncology Group/European Organisation for Research and Treatment of Cancer scoring. Survival analysis was performed using the Kaplan–Meier method. Differences in late toxicity and dose parameters between both groups were compared. Prognostic factors for survival and late toxicity were assessed using regression analyses.

**Results:**

Patients in the modified group developed late xerostomia and dysphagia less frequently than those in the comparison group did (*P* < 0.001). The mean dose (D_mean_) and V_26_ of parotid glands; D_mean_ and V_39_ of submandibular glands; and D_mean_ of sublingual glands, oral cavity, larynx, and superior, middle, and lower pharyngeal constrictor muscles were lower in the modified group than those in the comparison group (all *P* < 0.001). Both groups had no significant differences in overall, local recurrence-free, distant metastasis-free, or progression-free survival. The D_mean_ of the parotid and sublingual glands was a risk factor for xerostomia. The D_mean_ of the parotid and sublingual glands and middle pharyngeal constrictor muscle was a risk factor for dysphagia.

**Conclusions:**

Level IIb optimisation in NPC patients who meet certain criteria specially the exclusion of positive retropharyngeal nodes treated with intensity-modulated radiotherapy has the potential to better protect the salivary and swallowing structures, decreasing the development of late radiation-induced xerostomia and dysphagia while maintaining long-term survival.

**Supplementary Information:**

The online version contains supplementary material available at 10.1186/s12885-024-12391-7.

## Background

With the evolution of comprehensive treatment centred on intensity-modulated radiotherapy (IMRT), the survival rate of nasopharyngeal carcinoma (NPC) has notably improved, with a 10-year survival rate of 70–75% [1, 2]. In recent years, the focus of IMRT has shifted towards avoiding and reducing the long-term adverse effects of radiotherapy and improving the quality of life (QOL) of patients. Several landmark prospective multicentre randomised controlled trials have highlighted the potential for clinical target volume (CTV) optimisation. A multicentre study by Mao et al. [3] revealed that sparing the medial retropharyngeal lymph node region was non-inferior regarding local control while effectively preserving swallowing function. A separate randomised phase 3 trial by Tang et al. [4] demonstrated that elective irradiation of the uninvolved lower neck in patients with N0–N1 NPC did not negatively impact 3-year overall survival (OS), local recurrence-free survival (LRFS), and distant metastasis-free survival (DMFS). In addition, this approach reduces radiation toxicity.

Radiation-induced xerostomia is the predominant late toxicity of NPC radiotherapy, with an incidence rate between 60% and 90% [5, 6]. It causes many functional disorders in speaking, sleeping, chewing, tasting, swallowing, and oral health and even becomes a permanent condition that severely impairs patients’ QOL [7]. Dysphagia, a more pivotal determinant of QOL than xerostomia [8], can result in complications, such as gastroesophageal reflux [9], dehydration, malnutrition, cachexia, aspiration pneumonia, and even mortality. Dysphagia outperformed weight loss as a predictor of survival [10]. The evolution of IMRT has brought about an era of precision radiotherapy for NPC in which accurate target volume delineation and appropriate dose distribution are paramount for enhancing treatment efficacy and mitigating long-term toxicity [5, 11]. However, limited information is available regarding long-term survival outcomes and late toxicities in patients undergoing CTV-optimised treatment.

Our previous study suggested that reducing the upper border of level IIb in the CTV could reduce the dose to the parotid glands [12]. Therefore, this study further explored the effect of IIb CTV optimisation on the long-term survival of patients and investigated the protective effects on salivary and swallowing structures, as well as the potential preventive benefits in reducing the occurrence of late xerostomia and dysphagia.

## Methods

### Patients

This retrospective study included 415 patients newly diagnosed with NPC at our institution between December 2014 and October 2018. The inclusion criteria were: (1) pathologically confirmed nasopharyngeal squamous carcinoma (2), no prior treatment (3), no evidence of distant metastases at primary treatment (4), completion of the entire course of IMRT, and (5) no previous radiotherapy or head and neck surgery. The study was approved by the Ethics Committee of the Jiangsu Cancer Hospital. All study participants provided informed consent.

### Treatments

Computed tomography after administration of intravenous contrast medium was performed by collection of 3 mm slices from the head to the level of 2 cm below the sternoclavicular joint. The multimode image fusion technology was used as a reference to delineate the target area. Patients were immobilized in the supine position with a thermoplastic mask. All the patients received radical IMRT using simultaneous integrated boost (SIB) with 6 MV X-rays on a Varian Inspiration Platform (version 10.0) in our centre as reported previously [2]. And 9–11 radiation fields were used for the IMRT. The gross tumour volume (GTV) included the primary tumour (GTV1) and positive lymph nodes in the neck (GTV2). High-risk CTV1 was defined as the GTV plus a 5–10 mm margin containing the entire nasopharynx cavity and levels II and III cervical lymphatic drainage regions. The low-risk region was defined as CTV2, which encompassed CTV1 plus a margin of 5 mm, the lower neck without lymph node metastases, and the supraclavicular lymphatic drainage region, according to the guidelines of the Radiation Therapy Oncology Group (RTOG)/European Organisation for Research and Treatment of Cancer (EORTC) consensus delineations for NPC [11]. The planning target volume (PTV) was contoured by adding a 3 mm margin to the GTV and CTV. The prescribed radiation doses were as follows: total prescribed doses of 66–76 Gy/30–36 fractions were delivered to the PTV of GTV1; 66–72 Gy/32 fractions were delivered to the PTV of GTV2; 56–60 Gy/32 fractions were delivered to the PTV of CTV1; and 50–52 Gy/28 fractions were delivered to the PTV of CTV2. All patients were irradiated with 1 fraction daily, 5 days per week.

According to our institutional treatment protocol, patients with stage I disease underwent IMRT alone. For patients with stage II–IV disease, platinum-based chemotherapy was administered every three weeks before, during, or after radiation. Cumulative chemotherapy cycles were limited to no more than six throughout the period.

**Fig. 1:**
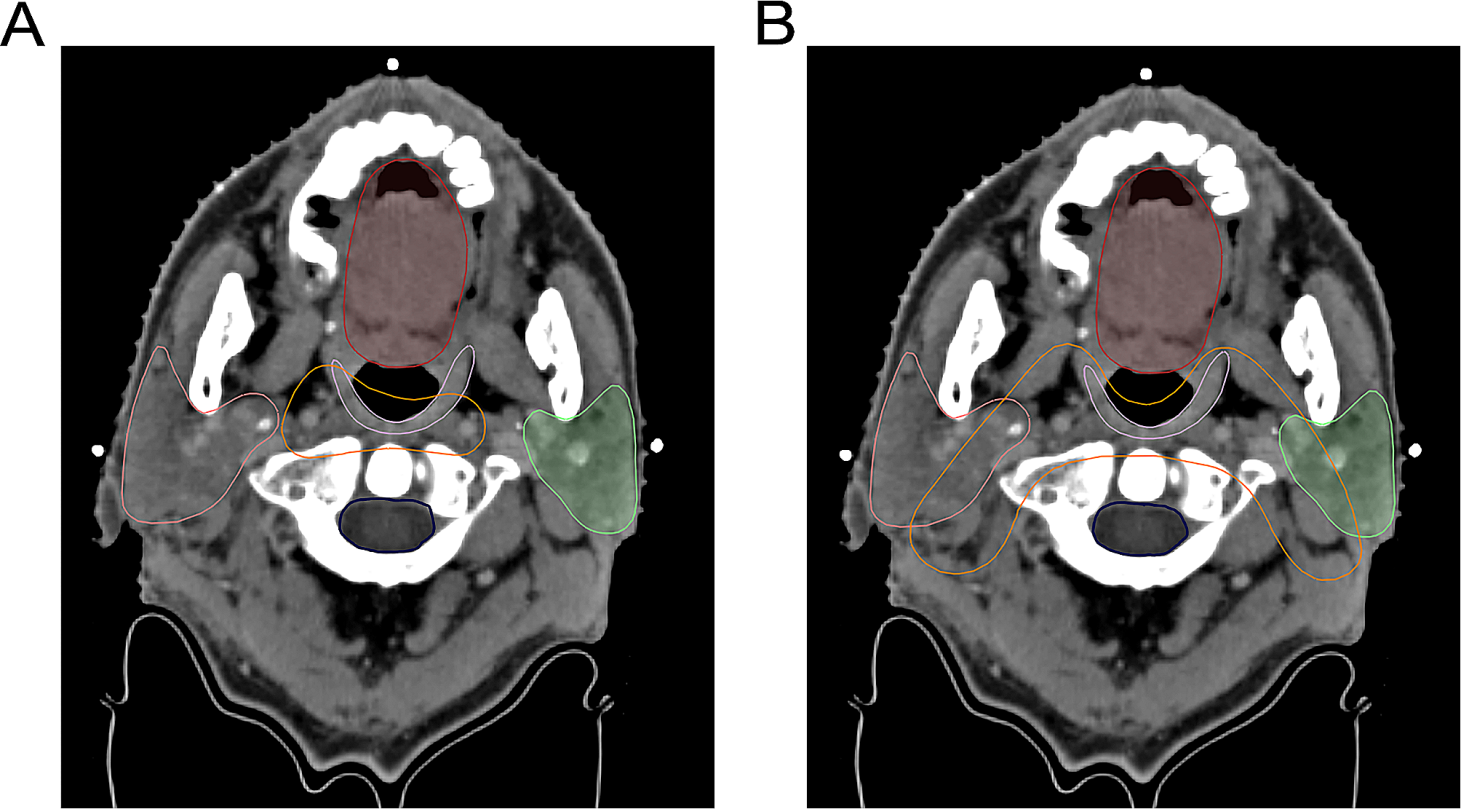
Level IIb delineation in two groups. **(A)** contouring method in the modified group; **(B)** contouring method in the comparison group. (yellow line: the CTV region; red line: the oral cavity; pink line: the right parotid gland; green line: the left parotid gland; purple line: the superior pharyngeal constrictor muscle; blue line: the spinal cord

**Fig. 2:**
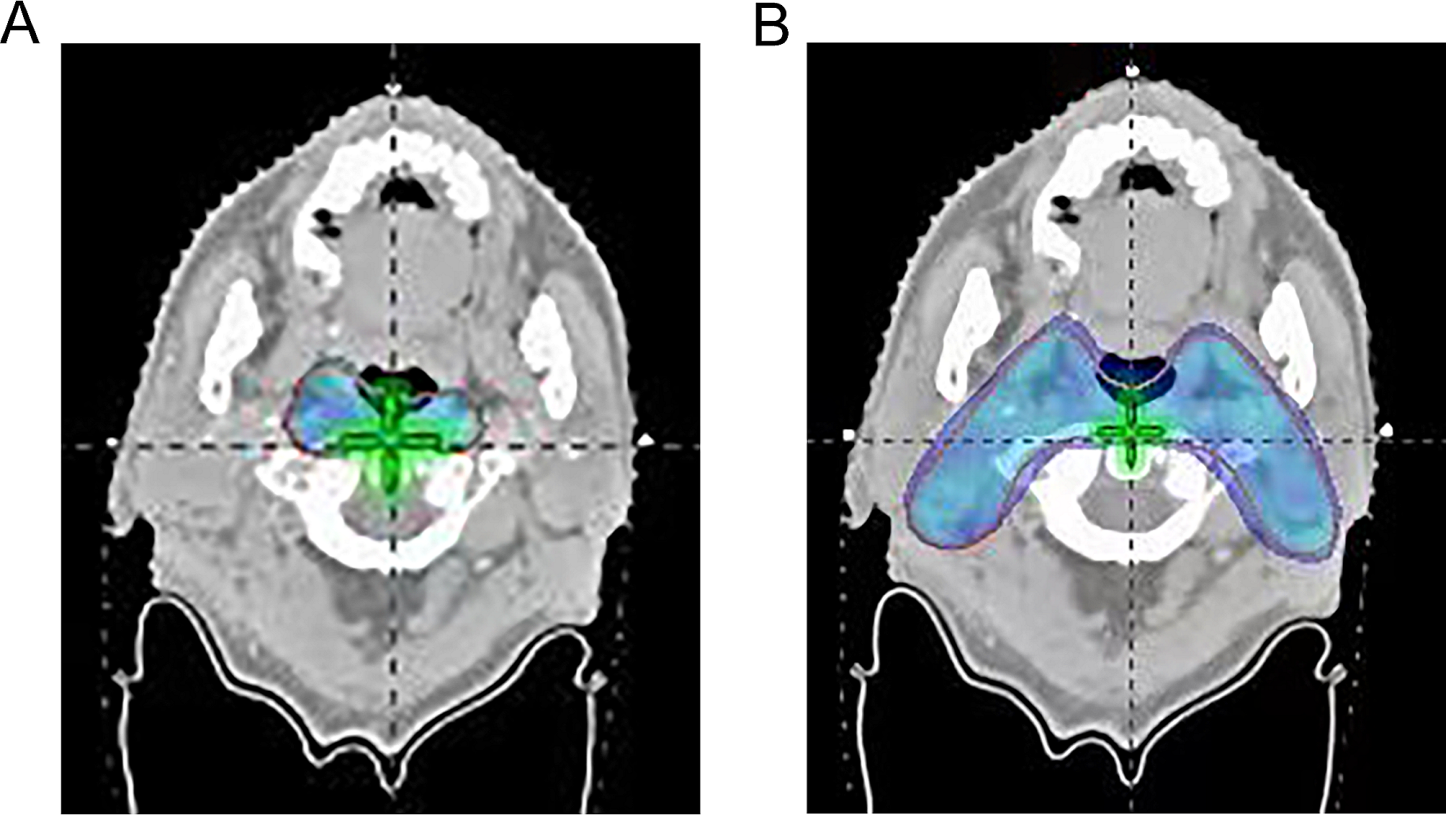
Dose distribution of 50 Gy in two groups. **(A)** dose distribution in the modified group; **(B)** dose distribution in the comparison group

The upper border of level IIb was delineated up to the skull base in the comparison group in accordance with the RTOG 0615 guidelines [13]. The upper border of level IIb in the modified group was delineated as the lateral process of the atlas. The difference in CTV delineation and dose distribution between groups were shown in Figs. 1 and 2, respectively. The patients who met the following criteria [12] were treated in the modified group: (1) the primary tumor demonstrated no expansion in the posterior or lateral directions on the ipsilateral side; (2) no positive retropharyngeal lymph nodes were present on the ipsilateral side; (3) on the ipsilateral side, the primary tumor did not invade the carotid sheath area, or did invade the carotid sheath area but demonstrated < 90° of invasion (the degree of contact arch between the tumor and carotid artery was less than 90°); (4) there was no positive lymph node in level II above the cranial edge of the second cervical vertebra (C2); (5) there was no visible lymph node in level II from the skull base to the upper edge of C2.

### Dosimetric assessment

Dose distributions to the salivary and swallowing structures were calculated using original radiotherapy treatment plans. The following structures were identified and delineated retrospectively in each participant’s actual IMRT plans: the bilateral parotid glands, bilateral submandibular glands, sublingual glands, oral cavity, larynx, and superior, middle, and lower pharyngeal constrictor muscles (PCMs). All structures were delineated by one radiation oncologist (with 5 years of experience in head and neck cancer), and the results were subsequently validated by a senior radiation oncologist (with 20 years of experience in head and neck cancer). Both professionals were blinded to the patient’s clinical data. All differences were resolved by discussion and both professionals were blinded to the clinical data of the patients. The oral cavity includes the surfaces of the inner lips, buccal mucosa, tongue, base of the tongue, floor of the mouth, and palate. The larynx is defined as the region extending from the superior edge of the epiglottis to the bottom of the cricoid. The contour of the superior pharyngeal constrictor includes the caudal tips of the pterygoid plates, extending through the upper edge of the hyoid bone. The middle pharyngeal constrictor was contoured from the upper to lower edge of the hyoid bone. The contour of the inferior pharyngeal constrictor extends below the hyoid bone to the inferior edge of the cricoid. The treatment planning system was employed to calculate the dose and volume histograms (DVHs) of each structure, measuring the mean dose (D_mean_) and V_x_ (volume of a structure receiving ≥ xGy) of the structures.

### Clinical follow-up

Weekly physical examinations and haematological tests were conducted during IMRT. Post-treatment follow-up was scheduled every 3 months for the first 1–2 years and every 6 months afterwards. Follow-up assessments included the evaluation of physical examination, relevant haematological tests, nasopharyngeal magnetic resonance image, chest and abdominal computed tomography scans, bone scans, and fibreoptic nasopharyngoscopy. The RTOG/EORTC late radiation toxicity scoring [14] was applied to evaluate the radiation-induced late xerostomia and dysphagia toxicity. The first evaluation of xerostomia and dysphagia began 12 months after the completion of radiotherapy and the evaluation was scheduled every 12 months. The last follow-up date was May 5th, 2023.

### Statistical analysis

Statistical analyses were performed using SPSS 26.0 software. OS, LRFS, DMFS, and progression-free survival (PFS) were calculated using the Kaplan–Meier method. We used the rank-sum test to compare xerostomia and dysphagia between the groups. The dose parameters of the structures were compared using independent-sample *t*-tests. For the univariate analysis, we used the log-rank test. The Cox proportional hazards model was used for multivariate analysis, and logistic regression was used to ascertain the risk factors for xerostomia and dysphagia. *P* < 0.05 was considered statistically significant.

## Results

### Patient baseline characteristics

A total of 415 patients were included in the study: 221 in the modified group and 194 in the comparison group. The baseline characteristics of the two groups were similar (Table 1). The median age of the entire cohort was 50 years (range, 14–81 years), and the male-to-female ratio was 3.66:1. The percentages of patients grouped as stages I, II, III, and IV were 2.6%, 20.2%, 39.0%, and 38.0%, respectively. The median radiation dose was 70 Gy (range, 66–80 Gy), and 376 (90.6%) patients underwent chemotherapy. Through the last visit (May 5th, 2023), the median follow-up durations for the whole cohort, modified group, and comparison group were 76, 74, and 85 months, respectively.

**Table 1:** Patient characteristics

	Modified group	Comparison group	*P* value
	*N* = 221	*N* = 194	
Age, mean (SD), years	49.86 (12.34)	49.55 (13.41)	0.648
Age, No (%)			0.209
< 50 years ≥ 50 years	98 (44.3) 123 (55.6)	98 (50.5) 96 (49.4)	
Sex, No (%)			0.700
Male	172 (77.8)	154 (79.3)	
Female	49 (22.1)	40 (20.6)	
Clinical stage^a^, No (%)			0.351
I II	8 (3.6) 44 (19.9)	3 (1.5) 40 (20.6)	
III IV	80 (36.1) 89 (40.2)	82 (42.2) 69 (35.5)	
T stage^a^, No (%)			0.398
T1	51 (23.0)	39 (20.1)	
T2	43 (19.4)	34 (17.5)	
T3	55 (24.8)	63 (32.4)	
T4	72 (32.5)	58 (29.8)	
N stage^a^, No (%)			0.205
N0	23 (10.4)	13 (6.7)	
N1	107 (48.4)	84 (43.2)	
N2	68 (30.7)	77 (39.6)	
N3	23 (10.4)	20 (10.3)	
Chemotherapy, No (%)			0.276
Yes	197 (89.1)	179 (92.2)	
No	24 (10.8)	15 (7.7)	
Radiotherapy dose, mean (SD), Gy	70.05 (4.60)	70.16 (2.92)	0.759

^a^According to the 8th edition of the UICC/AJCC staging workup

### Late xerostomia and dysphagia evaluation

Late xerostomia and dysphagia at the last follow-up visit are summarised in Table 2. The RTOG/EORTC late xerostomia grade 0, 1, 2, and 3–4 for the modified vs. comparison groups were 34.8% vs. 10.8%, 41.2% vs. 49.0%, 19.9% vs. 30.4%, and 4.1% vs. 9.8%, respectively. The RTOG/EORTC late dysphagia grade 0, 1, 2, and 3–4 for the modified vs. comparison groups were 70.6% vs. 27.8%, 19.9% vs. 47.4%, 9.0% vs. 23.2%, and 0.5% vs. 1.5%, respectively. Patients in the modified group developed late xerostomia and dysphagia less frequently than those in the comparison group did (*P* < 0.001).

**Table 2:** Comparison of RTOG/EORTC late xerostomia and dysphagia scores in the 415 cases

	Modified group	Comparison group	*P* value
RTOG/EORTC xerostomia, No (%)			
G0	77 (34.8)	21 (10.8)	< 0.001
G1	91 (41.2)	95 (49.0)	
G2	44 (19.9)	59 (30.4)	
G3–4	9 (4.1)	19 (9.8)	
RTOG/EORTC dysphagia, No (%)			
G0	156 (70.6)	54 (27.8)	< 0.001
G1	44 (19.9)	92 (47.4)	
G2	20 (9.0)	45 (23.2)	
G3–4	1 (0.5)	3 (1.5)	

Abbreviations: RTOG: Radiation Therapy Oncology Group; EORTC: European Organisation for Research and Treatment of Cancer

### Comparison of radiation dose parameters

We compared the D_mean_ and V_26_ of the bilateral parotid glands; D_mean_ and V_39_ of the bilateral submandibular glands; and D_mean_ of the sublingual glands, oral cavity, larynx, and superior, middle, and lower PCMs between both groups of patients. All radiation dose parameters in the modified group were lower than those in the comparison group (*P* < 0.001; Table 3).

**Table 3:** Comparison of the radiation dosimetric parameters of organs at risk in the 415 cases

OARs	Modified group	Comparison group	*P* value
Parotid D_mean_ (Gy)			
Left	31.20 ± 6.75	36.19 ± 6.36	< 0.001
Right	31.45 ± 6.50	35.88 ± 5.84	< 0.001
Parotid V_26_ (%)			
Left	53.75 ± 18.77	68.04 ± 16.24	< 0.001
Right	52.54 ± 18.94	67.11 ± 16.71	< 0.001
Submandibular D_mean_ (Gy)			
Left	41.42 ± 9.49	51.08 ± 8.60	< 0.001
Right	41.78 ± 10.07	51.74 ± 9.31	< 0.001
Submandibular V_39_ (%)			
Left	53.17 ± 28.13	81.79 ± 21.52	< 0.001
Right	53.03 ± 27.81	82.81 ± 22.31	< 0.001
Sublingual D_mean_ (Gy)	20.29 ± 3.59	30.90 ± 8.52	< 0.001
Oral cavity D_mean_ (Gy)	36.93 ± 4.03	43.63 ± 4.98	< 0.001
Larynx D_mean_ (Gy)	33.01 ± 5.15	39.25 ± 5.96	< 0.001
Superior PCM D_mean_ (Gy)	54.74 ± 8.22	62.52 ± 5.92	< 0.001
Middle PCM D_mean_ (Gy)	42.45 ± 6.98	50.03 ± 5.92	< 0.001
Inferior PCM D_mean_ (Gy)	36.88 ± 5.81	41.83 ± 6.45	< 0.001

Abbreviations: OAR: organs at risk; PCM: pharyngeal constrictor muscle

### Survival outcomes

**Fig. 3:**
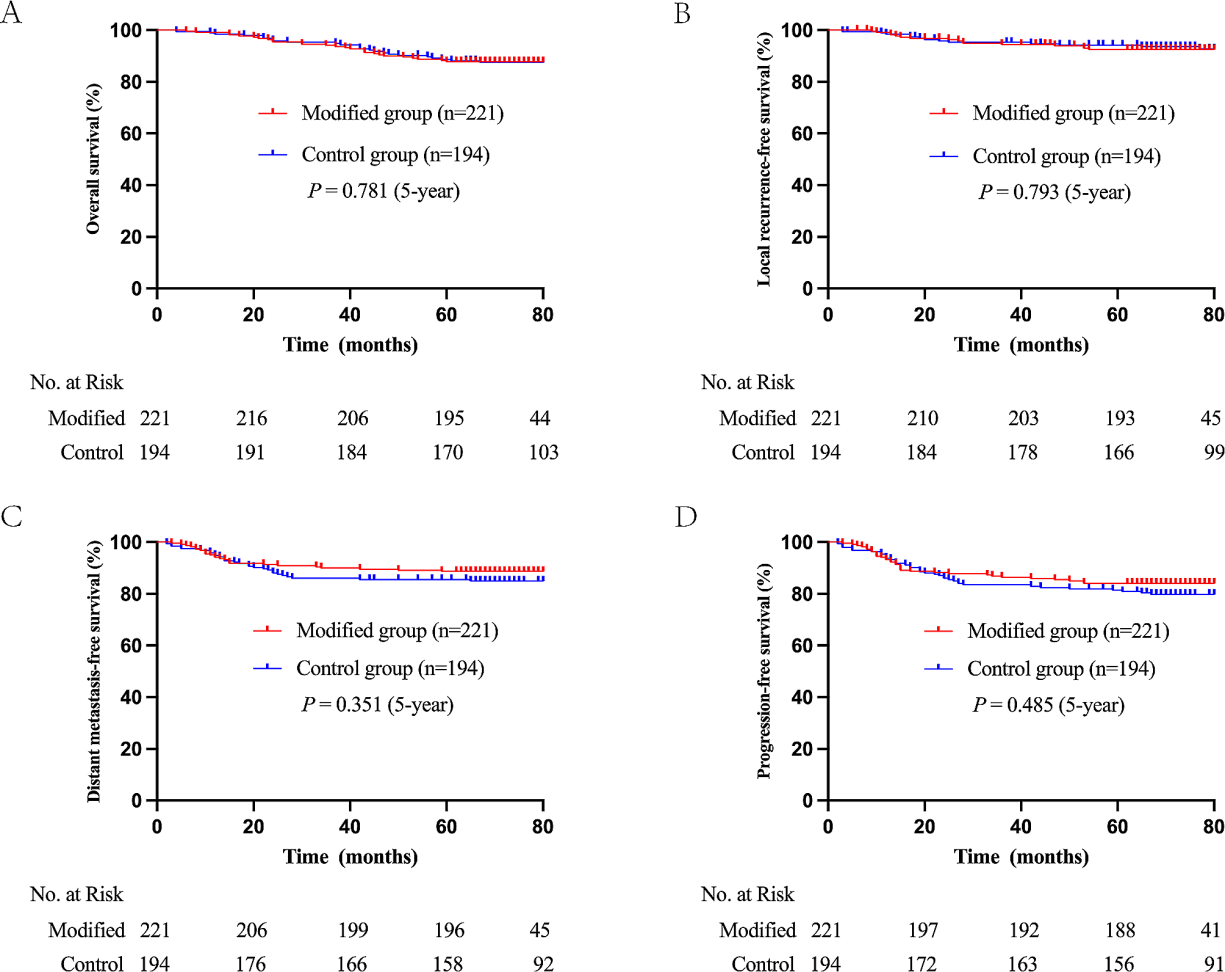
Kaplan–Meier curves for the modified and comparison groups in 415 patients. **(A)** overall survival; **(B)** local recurrence-free survival; **(C)** distant metastasis-free survival; **(D)** progression-free survival

The 5-year OS, LRFS, DMFS, and PFS rates were 88.2%, 93.4%, 87.2%, and 82.8%, respectively. The 5-year OS, LRFS, DMFS, and PFS rates in the modified vs. comparison groups were 87.8% vs. 88.7%, 92.7% vs. 94.3%, 88.6% vs. 85.5%, and 84.1% vs. 81.4%, respectively. There were no significant differences between the two groups (Fig. 3). Univariate and multivariate analyses were performed to identify independent prognostic factors for OS, LRFS, DMFS, and PFS, and the outcomes are presented in Supplementary Tables 1 and 2.

### Predictors of radiation-induced toxicities

The D_mean_ to the parotid, submandibular, and sublingual glands were included in univariate and multivariate analyses for late xerostomia. We included the D_mean_ to the parotid, submandibular, and sublingual glands; larynx; and superior, middle, and lower PCMs as variables in the univariate and multivariate analyses for late dysphagia. The multivariate analyses demonstrated that radiation dose parameters of the parotid glands (odds ratio [OR], 2.719; 95% confidence interval [CI], 1.768–4.182, *P* < 0.001) and sublingual glands (OR, 2.803; 95% CI, 1.822–4.311, *P* < 0.001) were risk factors for xerostomia symptoms; radiation dose parameters of the parotid glands (OR, 2.011; 95% CI, 1.266–3.192, *P* = 0.003), sublingual glands (OR, 2.343; 95% CI, 1.500–3.662, *P* < 0.001), and middle PCM (OR, 2.497; 95% CI, 1.576–3.958, *P* < 0.001) were risk factors for dysphagia symptoms (Table 4).

**Table 4:** Predictors of radiation-induced late toxicities

		Univariate analysis			Multivariate analysis		
Parameter	Variable^a^	Odds ratio	CI	*P* value	Odds ratio	CI	*P* value
Xerostomia	Parotid	4.447	3.128, 6.323	< 0.001	2.719	1.768, 4.182	< 0.001
	Submandibular	5.273	3.634, 7.650	< 0.001			0.125
	Sublingual	6.172	4.169, 9.138	< 0.001	2.803	1.822, 4.311	< 0.001
Dysphagia	Parotid	1.875	1.409, 2.496	< 0.001	2.011	1.266, 3.192	0.003
	Submandibular	1.724	1.299, 2.287	< 0.001			0.935
	Sublingual	1.889	1.420, 2.513	< 0.001	2.343	1.500, 3.662	< 0.001
	Larynx	1.568	1.187, 2.072	0.002			0.629
	Superior PCM	2.593	1.745, 3.852	< 0.001			0.629
	Middle PCM	2.000	1.498, 2.670	< 0.001	2.497	1.576, 3.958	< 0.001
	Lower PCM	1.364	1.036, 1.795	0.027			0.528

Abbreviations: PCM: pharyngeal constrictor muscle

^a^Univariate and multivariate analyses were calculated for each structure classified into high and low dose categories based on the median dose

## Discussion

Our research explored whether optimising the CTV of level IIb can sustain long-term survival and more effectively shield the salivary and swallowing structures to reduce the incidence of permanent xerostomia and dysphagia. There were no significant differences in OS, LRFS, DMFS, or PFS between the modified and comparison groups. Our study demonstrated robust long-term survival after optimising the CTV of level IIb in patients with a median follow-up duration of 76 months.

The salivary glands are comprised of the three pairs of major salivary glands (the parotid, submandibular, and sublingual) and numerous minor salivary glands throughout the oral cavity. The parotid glands primarily secrete predominantly serous saliva, whereas the sublingual and minor salivary glands secrete predominantly mucinous saliva. Submandibular glands produce mixed saliva, which contains both serous and mucinous components [15, 16]. The parotid glands predominantly contribute to stimulated salivary secretion. During sleep, the parotid glands rarely produce saliva, at which time, saliva is primarily secreted by the sublingual glands [17]. Eisbruch et al. [18] and Pointreau et al. [19] reported a gradual recovery of the parotid function after radiotherapy following the administration of radiation doses under 26 Gy to the parotid glands. Dijkema et al. [20] presented a definite normal tissue complication probability curve for parotid gland function in which no threshold dose was observed. However, mucin plays a crucial role in lubricating the oral structures, and protecting the parotid glands may be insufficient to alleviate xerostomia [21]. Stroom et al. [22] found that restricting the mean dose to the submandibular glands to below 39 Gy improved the salivary flow and reduced the symptoms of dry mouth.

In this study, all three pairs of major salivary glands, as well as minor salivary glands, were included to compare the bilateral parotid gland D_mean_ and V_26_, bilateral submandibular gland D_mean_ and V_39_, and the mean dose to the sublingual glands and oral cavity between the two groups. Our findings showed that the radiation dose to each salivary gland was reduced after CTV optimisation. Notably, the modified group exhibited significantly reduced occurrence of late xerostomia compared to that observed in the comparison group. Thus, CTV optimisation effectively safeguards the salivary glands and reduces the occurrence of late radiation-induced xerostomia while ensuring long-term survival. The multivariate analysis further highlighted that the mean dose to both the parotid and sublingual glands influenced late xerostomia, underscoring the role of mucin in preserving subjective oral moisture in patients [23].

Oropharyngeal swallowing is a complex and well-coordinated process consisting of three discrete phases. Food is processed into a bolus by mastication and salivary lubrication in phase 1 (the oral preparatory phase). In phase 2 (oral phase), the anterior oral tongue pushes the bolus back towards the oropharynx. The PCMs push the bolus, and the larynx closes the airway when the bolus is delivered towards the cervical oesophagus during phase 3 (pharyngeal phase) [8]. Disorders in any phase can lead to swallowing dysfunction, including damage to the swallowing structures and salivary glands. Feng et al. [24] confirmed a significant dose-volume effect relationship between aspirations and the mean dose to the PCMs and larynx receiving 50–65 Gy, which is also supported by a study by Charters et al. [25] Hedström et al. [26] definitively demonstrated the role of the D_mean_ to the contralateral parotid gland in predicting patient-reported dysphagia.

Our study evaluated the mean dose to the larynx and superior, middle, and lower PCMs, demonstrating that CTV optimisation resulted in less late dysphagia and reduced the radiation dose to swallowing structures. Furthermore, a significant association was observed between dysphagia and mean dose to the parotid gland, sublingual gland, and middle PCM.

A notable strength of this study relative to other clinical trials is its long follow-up duration (median, 76 months), which offers insights into long-term survival and late toxicity after radiotherapy. A long-term follow-up after CTV optimisation would undoubtedly provide more robust data and stronger validation. This study has some limitations. The data were retrospectively collected from a single centre and the inclusion criteria for the modified group has the potential to select a more favourable subgroup. Therefore, a potential bias may exist. The last follow-up xerostomia and dysphagia score was chosen for the toxicity endpoints which could vary among the patients. Mortality is a competing risk in the analysis. Further, non-dosimetric risk factors like smoking, chemotherapy and age were not considered in the multivariable analysis for toxicity. The comparative importance of the different organs at risk is uncertain since there is typically a high level of cross-correlation between DVHs of organs at risk. The results of our study should be further validated in prospective multicentre clinical trials.

## Conclusions

Our findings provide confident data suggesting that optimisation of level IIb has the potential to better protect salivary and swallowing structures and reduce the occurrence of late xerostomia and dysphagia while ensuring the long-term survival, which will benefit patients who meet certain criteria specially the exclusion of positive retropharyngeal nodes with non­metastatic NPC.

### Electronic supplementary material

Below is the link to the electronic supplementary material.

Supplementary Material 1

Supplementary Material 2

## Data Availability

The datasets used and/or analyzed during the current study are available from the corresponding author on reasonable request.

## Abbreviations

CTV: clinical target volume
NPC: nasopharyngeal carcinoma
IMRT: intensity-modulated radiotherapy
RTOG: Radiation Therapy Oncology Group
EORTC: European Organization for Research and Treatment of Cancer
PCM: pharyngeal constrictor muscles
QOL: quality of life
OS: overall survival
LRFS: local recurrence-free survival
DMFS: distant metastasis-free survival
GTV: gross tumour volume
OR: odds ratio
CI: confidence interval
