# Supplementary material for: Long-term follow-up of protective effects on salivary and swallowing structures and improvement of late xerostomia and dysphagia by level IIb optimisation in clinical target volume of nasopharyngeal carcinoma
Source: BMC Cancer. 2024 May 27;24:648. doi: 10.1186/s12885-024-12391-7 (PMC11129453; doi:10.1186/s12885-024-12391-7)
Supplement: Supplementary file 1 — Supplementary Material 1 [file 12885_2024_12391_MOESM1_ESM.docx]

Supplementary Table 1. Univariate analysis of prognostic factors

| Factors | OS (%) | *P* value | LRFS (%) | *P* value | DMFS (%) | *P* value | PFS (%) | *P* value |
| --- | --- | --- | --- | --- | --- | --- | --- | --- |
| Sex |  | 0.091 |  | 0.207 |  | 0.151 |  | 0.195 |
| Male | 86.5 |  | 92.9 |  | 86.1 |  | 80.9 |  |
| Female | 92.1 |  | 93.2 |  | 89.8 |  | 86.5 |  |
| Age |  | 0.507 |  | 0.735 |  | 0.967 |  | 0.916 |
| < 50 years | 87.7 |  | 91.8 |  | 87.7 |  | 83.1 |  |
| ≥ 50 years | 87.6 |  | 94.0 |  | 86.3 |  | 81.2 |  |
| T stage^a^ | | <0.001 |  | <0.001 |  | <0.001 |  | <0.001 |
| T1 | 96.6 |  | 93.3 |  | 97.7 |  | 92.2 |  |
| T2 | 89.6 |  | 92.2 |  | 87.0 |  | 79.2 |  |
| T3 | 93.2 |  | 94.9 |  | 90.6 |  | 87.2 |  |
| T4 | 75.3 |  | 91.5 |  | 76.1 |  | 72.3 |  |
| N stage^a^ | | 0.008 |  | <0.001 |  | 0.044 |  | 0.009 |
| N0 | 94.4 |  | 100 |  | 97.2 |  | 94.4 |  |
| N1 | 90.5 |  | 95.8 |  | 87.9 |  | 85.3 |  |
| N2 | 84.8 |  | 91.7 |  | 85.5 |  | 78.6 |  |
| N3 | 79.0 |  | 79.0 |  | 79.0 |  | 69.7 |  |
| Clinical stage^a^ |  | <0.001 |  | <0.001 |  | <0.001 |  | <0.001 |
| I | 100 |  | 100 |  | 100 |  | 100 |  |
| II | 96.4 |  | 96.4 |  | 95.2 |  | 91.6 |  |
| III | 92.5 |  | 94.4 |  | 90.7 |  | 85.8 |  |
| IV | 77.2 |  | 89.2 |  | 77.8 |  | 72.1 |  |
| Chemotherapy |  | 0.419 |  | 0.801 |  | 0.827 |  | 0.846 |
| Yes | 86.9 |  | 92.2 |  | 85.9 |  | 80.8 |  |
| No | 94.8 |  | 100 |  | 97.4 |  | 94.8 |  |
| Radiotherapy dose |  | 0.181 |  | 0.075 |  | 0.254 |  | 0.251 |
| ≥70 Gy | 87.5 |  | 93.5 |  | 86.6 |  | 81.0 |  |
| <70 Gy | 87.9 |  | 92.3 |  | 87.4 |  | 83.6 |  |

Abbreviations: OS, overall survival; LRFS, local recurrence-free survival; DMFS, distant metastasis-free survival; PFS, progression-free survival.

^a^According to the 8th edition of the UICC/AJCC staging workup.
