# Supplementary material for: Long-term follow-up of protective effects on salivary and swallowing structures and improvement of late xerostomia and dysphagia by level IIb optimisation in clinical target volume of nasopharyngeal carcinoma
Source: BMC Cancer. 2024 May 27;24:648. doi: 10.1186/s12885-024-12391-7 (PMC11129453; doi:10.1186/s12885-024-12391-7)
Supplement: Supplementary file 2 — Supplementary Material 2 [file 12885_2024_12391_MOESM2_ESM.docx]

Supplementary Table 2. Multivariate analysis of prognostic factors

| End-point | Factors | HR | 95% CI | *P* value^a^ |
| --- | --- | --- | --- | --- |
| OS | T stage |  |  | 0.250 |
|  | N stage |  |  | 0.350 |
|  | Clinical stage | 5.051 | 1.573, 16.217 | 0.006 |
| LRFS | T stage |  |  | 0.556 |
|  | N stage | 2.625 | 1.640, 4.203 | <0.001 |
|  | Clinical stage |  |  | 0.430 |
| DMFS | T stage | 1.875 | 1.394, 2.521 | <0.001 |
|  | N stage | 1.508 | 1.060, 2.146 | 0.023 |
|  | Clinical stage |  |  | 0.388 |
| PFS | T stage |  |  | 0.830 |
|  | N stage |  |  | 0.192 |
|  | Clinical stage | 2.108 | 1.496, 2.970 | <0.001 |

Abbreviations: OS, overall survival; LRFS, local recurrence-free survival; DMFS, distant metastasis-free survival; PFS, progression-free survival.

^a^Multivariate *P* values were calculated using Cox proportional-hazards model and the following parameters: T stage (T1–2 vs. T3–4), N stage (N0–1 vs. N2–3) and Clinical stage (stage I–II vs. stage III–IV).
